# A comparative study of robotic surgery and thoracoscopic surgery for mediastinal cysts

**DOI:** 10.1186/s12893-023-01994-9

**Published:** 2023-04-28

**Authors:** Ziqiang Hong, Yannan Sheng, Baiqiang Cui, Xiangdou Bai, Tao Cheng, Yingjie Lu, Xusheng Wu, Dacheng Jin, Yunjiu Gou, Jing Zhao

**Affiliations:** 1 The First Clinical Medical College of Gansu, University of Traditional Chinese Medicine, 35 East Dingxi Road, Lanzhou, Gansu, 730000 China; 2 First Department of Thoracic Surgery, Gansu Provincial Hospital, 204 Donggang West Road, Lanzhou, Gansu, 730000 China; 3 Lanzhou First People’s Hospital, No.1 West Street Wujiayuan, Lanzhou, Gansu, 730000 China

**Keywords:** Minimally invasive surgery, Robot-assisted thoracoscopic surgery, Video-assisted thoracoscopic surgery, Mediastinal cysts

## Abstract

**Objective:**

To compare the efficacy and safety of robotic-assisted thoracoscopic surgery (RATS) with video-assisted thoracoscopic surgery (VATS) in the treatment of mediastinal cysts.

**Methods:**

Retrospective analysis on clinical data of 70 cases of minimally invasive surgery for mediastinal cysts completed in the Department of Thoracic Surgery, Gansu Provincial People’s Hospital from April 2014 to December 2022. There were 34 cases in the RATS group with a cyst diameter of (3.70 ± 1.16) cm and 36 cases in the VATS group with a cyst diameter of (4.07 ± 1.20) cm. All cysts were evaluated preoperatively using magnetic resonance imaging (MRI) or chest computed tomography (CT) localization. Surgery-related indices were compared among the two groups.

**Results:**

All patients in two groups successfully completed resection of mediastinal cysts without perioperative deaths. Compared with the VATS group, the RATS group possessed shorter operative time [(75.32 ± 17.80) min vs. (102.22 ± 19.80) min, *P* < 0.001], lesser intraoperative bleeding [10 (5.00, 26.00) ml vs. 17.50 (5.00, 50.50) ml, *P* = 0.009], shorter postoperative chest drainage time [2 (1.00, 6.00) ml vs. 3 (2.00, 6.50) ml, *P* = 0.006] and shorter postoperative hospital stay [3 (2.00, 6.50) d vs. 4 (3.00, 7.50) d, *P* = 0.001]. There was no statistically significant discrepancy in intermediate openings and complications in both groups (*P* > 0.05).

**Conclusion:**

Compared with VATS, RATS is safety and effectivity in the treatment of mediastinal cysts and thus has advantages in operative time, intraoperative bleeding, postoperative chest drainage time and postoperative hospital stay.

## Background

Mediastinal cysts have low incidence and are usually asymptomatic. Most of them are found inadvertently during CT chest examinations for other diseases or routine physical examinations. Although mediastinal cysts are benign diseases, they may become complicated owing to infection, enlargement, spontaneous rupture and malignancy and some mediastinal cysts cannot be distinguished from mediastinal tumors before surgery [1]. Therefore, patients with suspicion should be surgically removed once they are detected.

Compared with traditional thoracotomy, VATS has the advantages of smaller incision, smaller trauma, fewer complications and rapider postoperative recovery [2–4]. It has been widely used in the treatment of mediastinal cysts. However, thoracoscope also has some inherent shortcomings, such as two-dimensional field of vision, insufficient processing capacity in narrow space (especially the upper mediastinum) and difficult operation such as suture and knotting. Due to the defects of thoracoscope, the Da Vinci robot-assisted surgery system emerged. Compared with VATS, RATS has been widely used for esophagectomy, lobectomy and other thoracic surgeries due to its advantages in automatic tremor filtering, three-dimensional field of view, 10-fold magnified images and 7 degrees of freedom of rotation [5–7], whereas the limited investigations on RATS for resection of mediastinal cysts have been reported. This study retrospectively analyzed the clinical data of 70 cases of minimally invasive surgery for mediastinal cysts completed at our center from April 2014 to December 2022, including 37 cases in the RATS group and 38 cases in the VATS group, to evaluate the safety and efficiency of RATS.

## Materials and methods

This study has been reviewed by the Ethics Committee of Gansu Provincial People’s Hospital (approval number: 2023-023) and was conducted in accordance with the national guide-lines and the Declaration of Helsinki. All patients signed the informed consent form for surgery before surgery.

### Clinical information

Inclusion criteria: (i) chest CT or MRI showed clear boundary between mediastinal cyst and surrounding tissue structure; (ii) postoperative pathology confirmed mediastinal cysts of different tissue origins; (iii) willing to undergo RATS or VATS.

Exclusion criteria: (i) poor cardiopulmonary function or severe arrhythmia cannot tolerate surgery; (ii) postoperative pathology confirms that it is not a cyst.

A total of 70 patients were included and either RATS or VATS was selected according to the patient’s intention and economic conditions, where 34 patients chose RATS and the remaining 36 patients selected VATS. The general information of the two groups was given in Table 1 and was comparable (*P* > 0.05).

### Surgery method

**Fig. 1:**
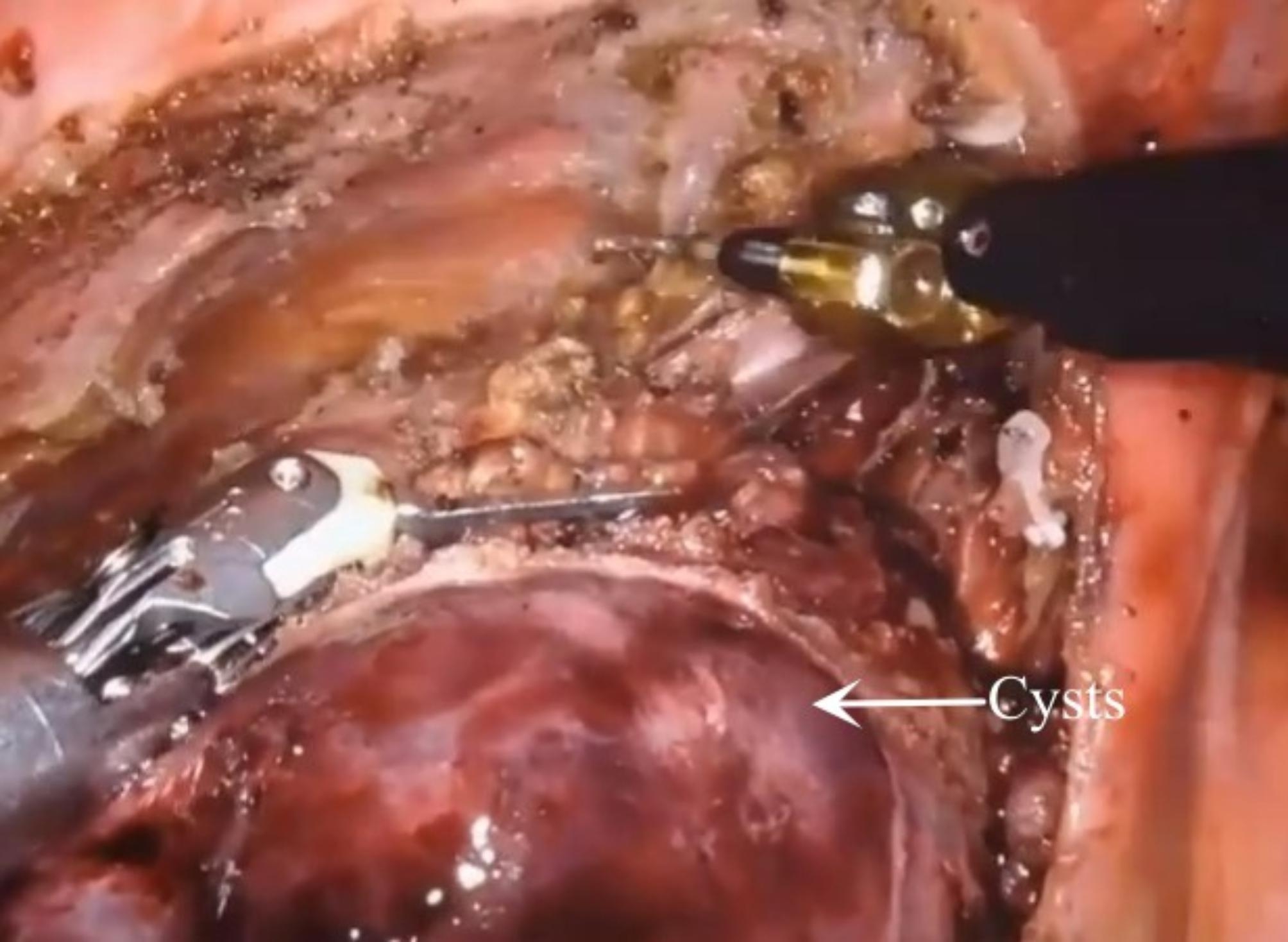
Intraoperative image

The RATS group used the da Vinci robotic surgical system (Si system) with a three-port approach and CO_2_ artificial pneumothorax. Position: Patients with anterior mediastinal cysts are placed in a 30-degree semi-supine position, exposing the ipsilateral axilla, while patients with middle and posterior mediastinal cysts are placed in a lateral position with a slight forward tilt to reduce the interference of lung tissue. Perforation position: For anterior mediastinal cysts, if the cysts locate right, the right thoracic approach is adopted, with the right anterior external body position and the hole position is set as “5-3-5” method, “5” is the observation hole, in the 5th intercostal space in the anterior axillary line of the affected side; “3” is the operation hole of the ② arm, in the 3rd intercostal space in the anterior axillary line; “5” is the operation hole of the ① arm, in the 5th intercostal space in the mid-clavicular line. If the cysts locate left-sided, the left thoracic approach is employed, with the left anterior external body position and the hole position is set as “5-3-5”. The posterior superior mediastinal cysts orifice is set as “6-4-7” method, “6” is the observation hole, which is between the 6th ribs in the posterior axillary line of the affected side; “4” is the operation hole of the ② arm, which is between the 4th ribs in the anterior axillary line; “7” is the operation hole of the ① arm, which is between the 4th ribs in the posterior axillary line. The “7” is the operation hole of the ① arm, which is located at the 7th intercostal space in the posterior axillary line. For the posterior inferior mediastinal cysts, the hole is set up in the “5-3-8” method. 3 intercostal space; “8” is the operation hole of the ① arm, in the 8th intercostal space of the posterior axillary line. The location of the lesion explored and the cyst is freed intact at the level of the outer layer of the intrinsic cysts envelope with an electrocoagulation hook. For larger thin-walled cysts with poor visualization, an additional auxiliary trocar (positioned optionally between the ribs in front of the entrance to the scope) or a suction device is fed into the trocar from the operating arm to puncture the cyst wall and aspirate the cyst fluid and then the cyst wall is removed with an electrocoagulation hook. The intraoperative picture is shown in Fig. 1. The specimen was removed from the operation hole of arm ①. Then the instruments and accessories were counted correctly, a chest drain was placed, the lung was expanded and the chest cavity was closed layer by layer.

A double-port VATS was used for anterior mediastinal cysts in the VATS group: a 3-cm incision was made at the 2nd or 3rd intercostal space in the anterior axillary line as an auxiliary operating port and the 5th intercostal space in the mid-axillary line as an observation port. For a mid-posterior mediastinal cyst, a single-port VATS is performed with the port set at the 5th intercostal space between the mid-axillary and posterior axillary lines.

### Observation indicators

The operative and postoperative data include operative time, intraoperative bleeding, postoperative chest drainage time, postoperative hospitalization time, transfer to open chest and postoperative complications, etc., on the basis of the medical record.

### Statistical analysis

SPSS 26.0 (SPSS Inc., Chicago, IL, USA) software was used for statistical analysis. If the measurement data conform to the normal distribution, it is expressed by mean ± standard deviation ($\overline x \pm s$) and the inter-group comparison is expressed by t-test. If the measurement data did not conform to the normal distribution, it is expressed by the median [M(P_25_, P_75_)] and the Man Whitney U test was used for comparison between groups. Categorical variables were described as frequencies and percentages (%) and the chi-square test or Fisher test was applied to compare the results among groups. Herein, *P* < 0.05 was considered to be statistically significant difference.

## Results

Both groups successfully completed the surgery without perioperative deaths. There was no intermediate open chest in the RATS group and one case in the VATS group (injury to the left innominate vein). Compared with the VATS group, the RATS group has shorter operative time (*P* < 0.001), lesser intraoperative bleeding (*P* = 0.009), shorter postoperative chest drainage time (*P* = 0.006) and shorter postoperative hospital stay (*P* = 0.001). There was no statistically significant difference in the rate of transferring to open chest and postoperative complication rate between the two groups (*P* > 0.05), as listed in Table 2.

Pathological findings: all postoperative pathologies confirmed cysts of different histological origins and the results of classifying them according to pathological types are shown in Table 1. Patients in both groups were successfully followed up after surgery without lost cases and both groups recovered well without recurrence.

**Table 1:** General information of patients [cases(%)/$\overline x \pm s$]

Characteristic	RATS group (n = 34)	VATS group (n = 36)	*P*
Sex			0.611
Male	14 (41.2)	17 (47.2)	
Female	20 (58.8)	19 (52.8)	
Age (years)	41.03 ± 7.38	43.06 ± 6.82	0.237
BMI (kg/m^2^)	23.41 ± 4.12	23.28 ± 3.75	0.887
Cyst diameter (cm)	3.70 ± 1.16	4.07 ± 1.20	0.198
Cyst location			0.734
Anterior mediastinum	23 (67.6)	26 (72.2)	
Middle mediastinum	5 (14.8)	6 (16.7)	
Posterior mediastinum	6 (17.6)	4 (11.1)	
Symptoms			0.950
No	17 (50.0)	19 (52.8)	
Chest Pain	4 (11.8)	2 (5.6)	
Chest tightness	6 (17.7)	7 (19.4)	
Cough	3 (8.8)	5 (13.8)	
Dyspnea	1 (2.9)	1 (2.8)	
Eating difficulties	1 (2.9)	1 (2.8)	
Fever	2 (5.9)	1 (2.8)	
Pathological type			0.858
Bronchogenic cysts	16 (47.1)	15 (41.7)	
Thymic cysts	13 (38.2)	16 (44.4)	
Pericardial cysts	4 (11.8)	3 (8.3)	
Esophageal cysts	1 (2.9)	2 (5.6)	

BMI, body mass index

**Fig. 2:**
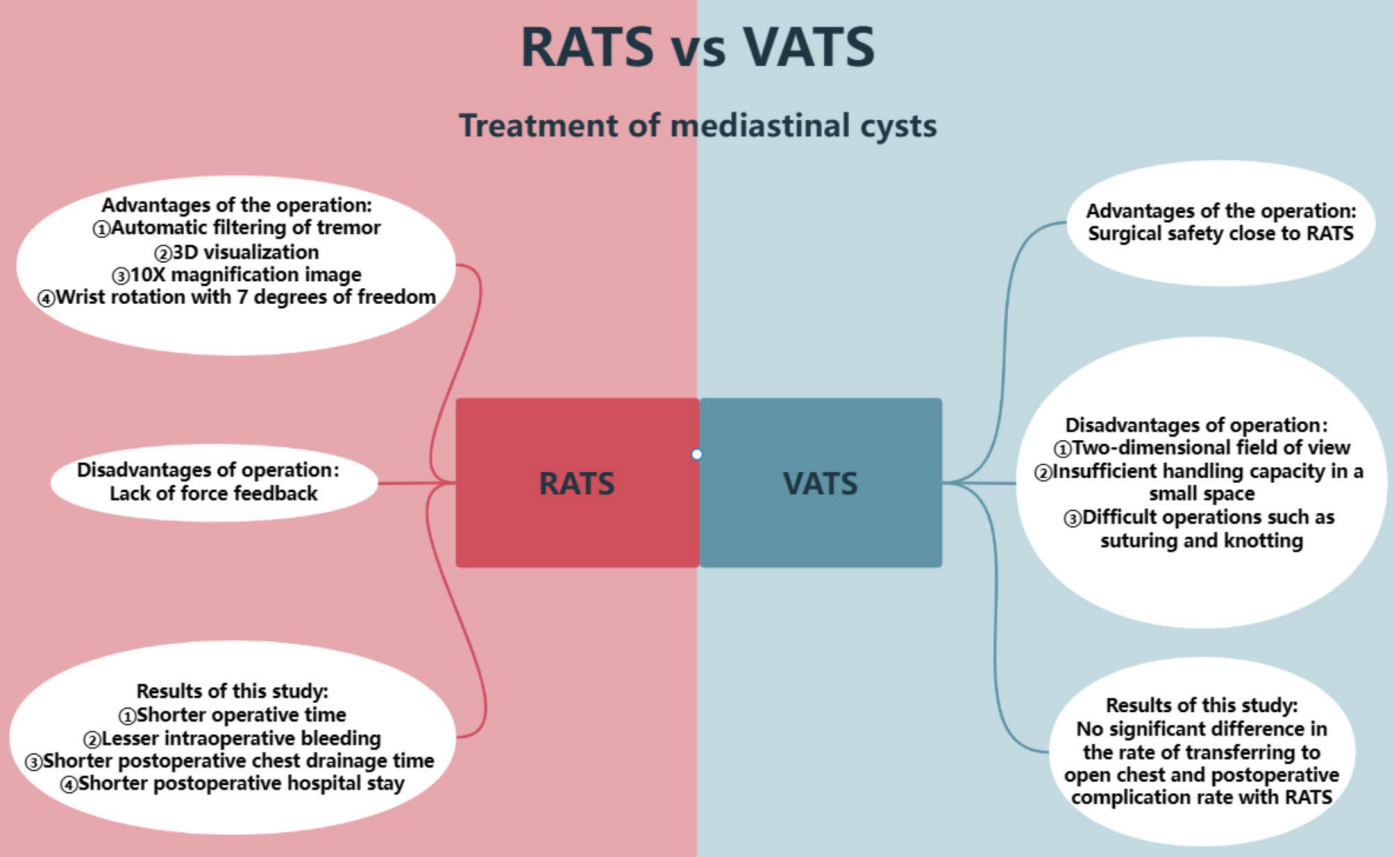
A graphical abstract

## Discussion

Mediastinal cysts are benign lesions due to congenital developmental anomalies and commonly include bronchial cysts, thymic cysts, esophageal cysts and pericardial cysts [8]. Bronchial cysts were the most common lesions among the patients included in this study (44.29%). The presence or absence or severity of symptoms in patients are related to the size of the cyst, the site of occurrence and the degree of organ compression and the probability of clinical symptoms is higher in bronchogenic cysts among various cysts. When cystic lesions are small, patients usually have no clinical symptoms and are mostly found during physical examination; 51.43% of patients in our group were found during physical examination. Although benign, mediastinal cysts tend to increase in size. When the lesion enlarges, it is prone to compress symptoms such as dyspnea, dysphagia, hoarseness and superior vena cava syndrome [9] and may also present with chest tightness, cyanosis, fever, coughing sputum and hemoptysis. These symptoms were manifested in most of the patients in this study, including 6 patients with chest pain, 13 with chest tightness, 8 with cough, 2 with dyspnea, 2 with difficulty in eating and 3 with fever.

The preoperative diagnosis of mediastinal cysts mainly relies on imaging examinations such as chest CT and MRI, among which MRI is of the highest accuracy rate; ultrasound examination can clarify whether the mass is cystic or solid; bronchoscopy, bronchography, aortography, barium esophagogram and cyst puncture are beneficial for differential diagnosis [10]. A definitive diagnosis still requires postoperative pathological examination. Mediastinal cysts are difficult to diagnose preoperatively and asymptomatic individuals may develop severe symptoms over time secondary to infection, bleeding, rupture and malignancy [11]. Surgery becomes more difficulty when symptomatic and complications are more frequent, especially in bronchogenic cysts, which in children are relatively soft and susceptible to compressional stenosis due to the tracheobronchial tree and usually occupy the space needed for normal tissue development. The indications for surgery of mediastinal cysts in the presence of clinical symptoms are clear, but the indications and timing of surgery in asymptomatic patients are inconclusive. Most scholars believed that mediastinal cysts indicated the immediate surgery once they were detected. Mediastinal cysts are benign diseases and most lesions are stable; at the same time, advances in minimally invasive techniques allow surgery to be performed under VATS or RATS in the majority of cases. Therefore, we believe that for patients without obvious clinical symptoms, imaging easily distinguishable from solid tumors such as mediastinal tumors, small cyst diameter and no growth trend, regular review follow-up can be chosen, especially for elderly patients with poor surgical tolerance.

**Table 2:** Surgical data of patients [cases(%)/$\overline x \pm s$]

Characteristic	RATS group (n = 34)	VATS group (n = 36)	*P*
Operation time (min)	75.32 ± 17.80	102.22 ± 19.80	< 0.001
Intraoperative bleeding volume (ml)	10 (5.00, 26.00)	17.50 (5.00, 50.50)	0.009
Postoperative chest drainage time (d)	2 (1.00, 6.00)	3 (2.00, 6.50)	0.006
Postoperative hospital stay (d)	3 (2.00, 6.50)	4 (3.00, 7.50)	0.001
Transfer to open chest	0	1 (2.8)	0.328
Postoperative complications	2 (5.9)	3 (8.3)	0.182
Pulmonary infection	1 (2.9)	0	
Chylothorax	0	1 (2.8)	
Hoarseness	0	1 (2.8)	
Arrhythmia	1 (2.9)	1 (2.8)	
Follow-up time (month)	26.29 ± 6.39	28.19 ± 6.94	0.238

Note: The procedure time for the RATS group is the time from skin incision to suture, including the docking time

The results of this study showed that the RATS group has shorter total operative time, lesser intraoperative bleeding, shorter postoperative chest drainage and shorter postoperative hospital stay compared with the VATS group. Although there is additional loading time for RATS, the total procedure time for RATS is shorter because of the clearer intraoperative view and easier maneuvering in tight spaces. In addition, with the experience of the operator and the skillful cooperation of the robotic team, the operation time of RATS can be significantly reduced. Due to the more delicate intraoperative operation of RATS, the patient’s normal tissues are better protected and the trauma of patients is smaller, so the recovery of patients after operation is also faster.

The cyst should be carefully peeled off during surgery and the cyst wall should be removed completely [12]; incomplete resection may result in serious complications such as esophageal, pleural, bronchial and skin fistulae and recurrence [13]. If complete excision is impossible due to extensive adhesions, the residual cyst wall can be electrocautery to prevent recurrence. If the tip is attached to the mediastinal tissue, it should be cut off immediately against the mediastinal tissue. When the cyst is large, in order to avoid damaging the neighboring tissues, the cyst can be incised first and the contents can be aspirated and then the cyst wall can be removed, taking care to avoid contaminating the chest cavity with the contents. In 10 patients in this group, due to the large size of the cyst or the location of the lesion affecting the operative field, the contents were first aspirated with a syringe and then the cyst wall was excised; for those who could not be aspirated, the cyst was excised after the contents were released by incision. When excising the cyst wall, attention should be paid to the relationship between trachea, bronchus and esophagus and when it is closely adhered to the above structures, it should be carefully peeled off and if necessary, the corresponding structures can be excised and necessary repairs can be made.

Thoracoscopy and instruments have inherent defects and are more difficult to remove cysts in narrow spaces such as vascular spaces. In a case series of 84 patients who underwent RATS resection of mediastinal masses, Chen et al. pointed out that RATS incorporates three-dimensional imaging, removes hand tremors, and has a wider range of surgical instrument motion [14]. Combined with our experience and experience in implementing RATS for the treatment of thoracic tumors, we found that the advantages of RATS can be better utilized in the treatment of mediastinal cysts in confined spaces. The specific advantages of RATS can be demonstrated as follows: (i) the magnified three-dimensional field of view can magnify the structures in the narrow space of the mediastinum; (ii)flexible and multi-angle “wrist” can avoid the important tissues and organs; (iii) solving the problem of “straight” conventional lumpectomy instruments; (iv) surgeon’s comfort leads to the safety of patient surgery; (v) It enabled surgeons to gain a longer career life.

When operating with the da Vinci robotic surgical system, we routinely establish an artificial pneumothorax of 8 mm Hg, which can largely increase the pleural cavity space on the operative side, especially in the anterior mediastinal region, with its narrow potential space, poor exposure and many important vascular structures. Due to the establishment of an artificial pneumothorax, the potential space of the anterior mediastinum can be effectively enlarged and the above-mentioned advantages of the robotic surgical system, the surgical safety is greatly improved and thus the surgical operation is more comfortable when the cyst is resected with the robotic surgical system, so the mediastinal cyst resection should be best option for the da Vinci robotic surgical system. However, after the establishment of the artificial pneumothorax, some of the small cysts in the anterior mediastinum may be pushed to the opposite side and not in the position shown on the imaging and the lesions may be missed because of the narrow field of view due to the magnification of the robotic field of view, which should be carefully explored intraoperatively.

Our present study is of some limitations and shortcomings: (i) possible bias in the results due to the single-center data source of the included studies; (ii) smaller sample sizes may lead to selection bias.

## Conclusion

In summary, this study shows that RATS is safe and feasible in the treatment of mediastinal cysts, with better short-term efficacy than VATS and RATS can be strongly promoted in the treatment of mediastinal cysts. The advantages and disadvantages of RATS versus VATS and the comparison of surgical results are shown in Fig. 2. We believe that as we become more proficient in using the da Vinci robotic surgical system, the system will show the greater advantages of use and demonstrate its increased application in the field of thoracic surgery.

## Data Availability

The datasets used and/or analysed during the current study are available from the corresponding author on reasonable request.

## Abbreviations

RATS: Robot-assisted thoracoscopic surgery
VATS: Video-assisted thoracoscopic surgery
MRI: Magnetic resonance imaging
CT: Computed tomography
BMI: Body mass index
